# A data-driven acute inflammation therapy

**DOI:** 10.1186/1755-8794-6-S3-S7

**Published:** 2013-11-11

**Authors:** Vladan Radosavljevic, Kosta Ristovski, Zoran Obradovic

**Affiliations:** 1 Center for Data Analytics and Biomedical Informatics, Temple University, Philadelphia, PA 19122, USA

## Abstract

Acute inflammation is a severe medical condition defined as an inflammatory response of the body to an infection. Its rapid progression requires quick and accurate decisions from clinicians. Inadequate and delayed decisions makes acute inflammation the 10th leading cause of death overall in United States with the estimated cost of treatment about $17 billion annually. However, despite the need, there are limited number of methods that could assist clinicians to determine optimal therapies for acute inflammation. We developed a data-driven method for suggesting optimal therapy by using machine learning model that is learned on historical patients' behaviors. To reduce both the risk of failure and the expense for clinical trials, our method is evaluated on a virtual patients generated by a mathematical model that emulates inflammatory response. In conducted experiments, acute inflammation was handled with two complimentary pro- and anti-inflammatory medications which adequate timing and doses are crucial for the successful outcome. Our experiments show that the dosage regimen assigned with our data-driven method significantly improves the percentage of healthy patients when compared to results by other methods used in clinical practice and found in literature. Our method saved 88% of patients that would otherwise die within a week, while the best method found in literature saved only 73% of patients. At the same time, our method used lower doses of medications than alternatives. In addition, our method achieved better results than alternatives when only incomplete or noisy measurements were available over time as well as it was less affected by therapy delay. The presented results provide strong evidence that models from the artificial intelligence community have a potential for development of personalized treatment strategies for acute inflammation.

## Introduction

Acute inflammation is a progressively severe medical condition defined as an inflammatory response of the body to a trauma, a surgery, a burn, or an infection. Fast progression of acute inflammation requires quick and accurate decisions from clinicians. Inadequate and delayed decisions make acute inflammation the 10th leading cause of death overall in United States, with the estimated cost of treatment about $17 billion annually [1]. However, despite the need, there are no computational methods that can help clinicians in planning optimal therapies for acute inflammation.

Model predictive control (MPC) is a method often used in clinical applications for planing of optimal treatment [2-5] mainly due to its inherent capability to handle clinically relevant constraints and to take into account multiple variables at the same time. A predictive model is the most important part of MPC, which is deployed to estimate patient's response to the therapy. These estimations together with a set of clinically relevant constraints are used in MPC to compute optimal therapies that will lead the patient to the healthy condition. The efficacy of MPC based treatment is completely dependent on model's capability to accurately estimate the patient's response to therapy.

To construct an accurate predictive model, practitioners often rely on domain-based assumptions about patients' behavior. Such a domain-driven model was previously used as a component in MPC to find optimal acute inflammation treatment [6]. The authors showed that pro-inflammatory and anti-inflammatory medications within MPC setup decreased the mortality rate. However, therapy outcome was greatly dependent on the set of parameters used in the model.

To relax the dependency on parameter settings, a data-driven predictive model that learns patients' responses from observed data and doesn't rely on any domain-based hypothesis can be used instead. MPC with data-driven predictive models was successfully applied in medical applications, including optimal glucose control [7], the control of anticancer medications dosage [8], the control of ventilation [9], and finding an adequate dosage of anesthesia [10]. The main objective of this study is to show that MPC with a data-driven predictive model can improve the overall quality of acute inflammation treatments.

Our preliminary results [11] indicated that a data-driven MPC method significantly improved the number of healthy outcomes when compared to acute inflammation therapy strategies from literature and clinical practice. This paper reports a more detailed study by evaluating the data-driven MPC and alternative approaches over different clinically relevant setups as follows. All experiments are done on virtual patients generated by a mathematical model that emulates inflammatory response, which is a standard approach in pharmacological research. We show that in achieving results presented in [11], our method used much lower doses of medications than other methods. Furthermore, our method provides good accuracy even in the presence of incomplete measurements, as well as additive Gaussian noise. It is also less affected by possible therapy delay than the alternatives. We also identified characteristics of patients who may benefit the most from application of our method. Consequently, our data-driven tool for the optimal treatment of acute inflammation along with domain-based knowledge may provide a platform for the development of personalized treatment strategies that will increase survival rates.

The rest of the paper is organized as follows. In the next section we introduce a virtual patient model that emulates therapy response and we define clinically relevant therapy constraints. Then we propose a data-driven predictive model that determines optimal therapy, which is followed by evaluation of the model. In the final section we give a conclusion.

## Mathematical model for virtual patient and treatment constraints

Virtual patients are carefully developed mathematical models made to mimic the human body behavior when exposed to circumstances that are in the interest of a study. They allow biomedical researchers to perform various types of experiments on the same patient as well as to compare the outcomes. Thus, the main purpose of having virtual patients is to reduce expense for clinical trials and to lower chance of failures. A mathematical model in the form of the system of ordinary differential equations (ODE), recently proposed in [6], simulates inflammatory response to an infection, including medication effect on inflammatory response. It models the dynamics of concentration of:

• bacterial pathogen (*P*),

• early pro-inflammatory mediators (*N*),

• tissue damage markers (*D*),

• anti-inflammatory mediators (*CA*),

which are regulated by anti-inflammatory (*AIDOSE*) and pro-inflammatory (*PIDOSE*) medications via following equations

(1) $$\frac{dP}{dt}=k_{pg}\left(1-\frac{P}{P_{\infty }}\right)-\frac{k_{pm}s_{m}P}{\mu_{m}+k_{mp}P}-k_{pn}f\left(N\right)P,$$

(2) $$\frac{dN}{dt}=\frac{s_{nr}R}{\mu_{nr}+R}-\mu N+PIDOSE\left(t\right),$$

(3) $$\frac{dD}{dt}=\frac{k_{dn}f{\left(N\right)}^{6}}{x_{dn}^{6}+f{\left(N\right)}^{6}}-\mu_{d}D,$$

(4) $$\frac{dCA}{dt}=s_{c}+\frac{k_{cn}f\left(N+k_{cnd}D\right)}{1+f\left(N+k_{cnd}D\right)}-\mu_{c}CA+AIDOSE\left(t\right),$$

where

(5) $$R=f\left(k_{np}P+k_{nn}N+k_{nd}D\right),$$

(6) $$f\left(x\right)=\frac{x}{1+{\left(\frac{CA}{c_{\infty }}\right)}^{2}}.$$

Mathematical model in this form is capable of modeling the complex cascade of inflammation initiated by pathogen (*P*). An increase of pathogen level *P *leads to the series of positive and negative feedback reactions that are all successfully modeled by ODE. In particular, an increase of *P *causes the development of a pro-inflammatory response (the increase of *N*) and the development of tissue damage (the increase of *D*). Equation (1) simulates a positive effect of inflammation, where an increase of *N *reduces level of pathogen *P*. However, (3) simulates a negative effect of inflammation, where an increase of *N *further damages tissue causing rapid increase of *D*. An increase of *D *activates a negative feedback in (4), or anti-inflammatory response (*CA*), which lowers the level of *N *and prevents tissue damage (decrease of *D*) [6]. The strength of positive and negative feedbacks depends on the parameter values in ODE. By varying parameter values we can simulate variability among patients.

**Figure 1:**
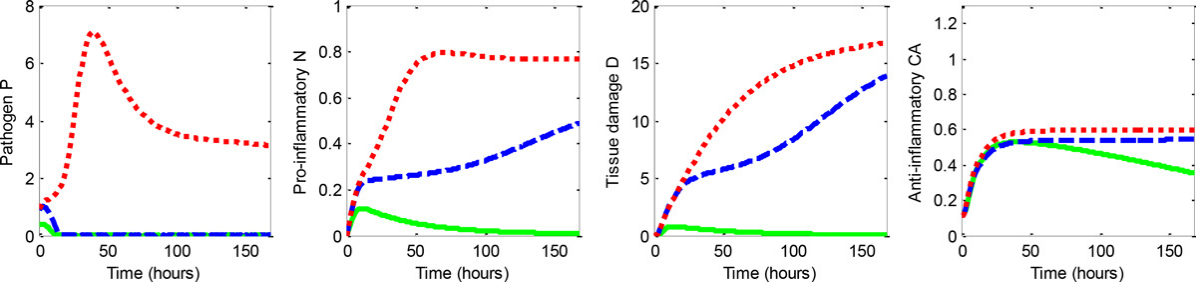
**Progress over time of pathogen (*P*), pro-inflammatory mediator (*N*), tissue damage (*D*), and anti-inflammatory mediator (*CA*) for patients with septic (red), aseptic (green), and healthy (blue) outcomes when no therapy was applied**.

A diverse population of patients is generated by random initialization of parameters *k_pg_*, *k_cn_*, *k_nd_*, and initial conditions *P*_0 _and *CA*_0 _from uniform distribution on valid ranges (*k_pg _*∈ [0.3, 0.6], *k_cn _*∈ [0.03, 0.05], *k_nd _*∈ [0.015, 0.025], *P*_0 _∈ [0, 1], *CA*_0 _∈ [0.0938, 0.1563]). Other parameters were set to constant values as in [6] except *k_cnd _*that covaries with *k_cn _*and *k_np _*that covaries with *k_nd _*[6]. Patients in all simulations are observed in hourly steps *t*, starting from *t *= 0 when parameters and patient state are initialized. Then, patient state changes over time following ODE for 168 hours (one week) when simulation is over. As in [6], we consider three possible outcomes depending on the patient state [*P N D CA*] at the end of simulation time (Figure 1):

• healthy (*P <*1, *N <*0.05, *D <*1),

• aseptic death (*P <*1, *N >*0.05, *D >*1),

• septic death (*P >*1, *N >*0.05, *D >*1).

Evolution of the patient to the final state can be modulated by carefully constrained pro-inflammatory (*PIDOSE*) and anti-inflammatory (*AIDOSE*) medications. Therefore, model predictive control is applicable to inflammation therapy only if constraints on medication doses obey clinical rules. Oppositely, for example, a large amount of medication given at once can cause the death of a patient due to overdose. Also, the prescription of a high level of anti-inflammatory doses with long duration may predispose the patient to other infections, which might cause death. We follow well-defined medication constraints for inflammation treatment stated in [6]:

• $0\leq PIDOSE\leq PIDOSE_{{}^{k}}^{MAX}$, where $PIDOSE_{k}^{MAX}$ is the difference between *N_max _*(*N_max _*= 0.5) and the current level of *N *= *N_k_*;

• $0\leq AIDOSE\leq AIDOSE_{{}^{k}}^{MAX}$, where $AIDOSE_{k}^{MAX}$ is the difference between a maximum allowable level of *CA *(initialized to *CA_max _*= 0.6264) and the current level of *CA *= *CA_k_*;

• *saturation of anti-inflammatory mediator*: the saturation of CA for long durations is avoided in clinical practice because of other infections that can occur and endanger organ recovery. Thus, if the level of *CA *has been elevated for 48 hours, then *CA_max _*was halved.

## Model predictive control (MPC)

**Figure 2:**
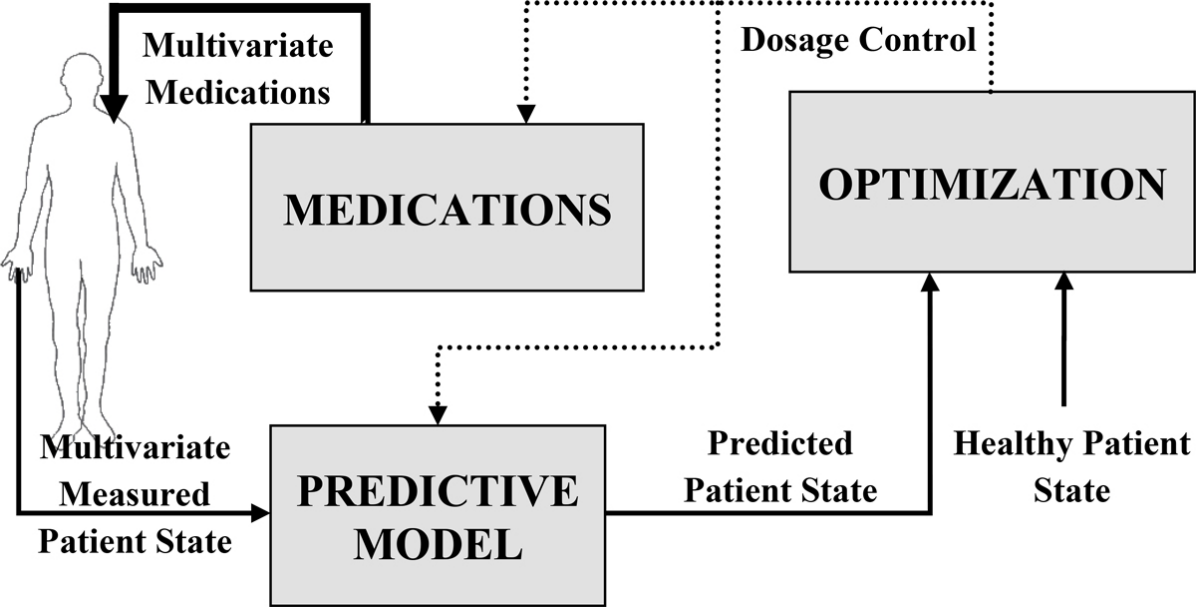
**Block scheme of model predictive control strategy for finding optimal medication doses**.

Model predictive control finds an optimal set of control inputs by minimizing an objective function while preventing violations of predefined constraints. The objective function is based on the difference between outputs of the predictive model and reference trajectory. In the case of inflammation therapy, when the reference trajectory is set to *D *= 0 and *P *= 0, MPC finds the set of medication doses which would lead the patient to a healthy state [6] (see Figure 2). In order to find medication doses at certain time point *k*, we need to define following:

• *prediction horizon *(*p*) is the time window that governs how many future outputs of the predictive model will be used in the objective function;

• *predicted patient states *$\left\{{\left[\hat{P}\;\hat{N}\;\hat{D}\;ĈA\right]}_{k+j},j=\text{ 1},\;.\;.\;.\;,\;p\right\}$ are estimations of output variables obtained from the predictive model;

• *control horizon *(*c*, *c ≤ p*) is the time window that governs how many future controls (medication dosage) will be determined by the optimization algorithm;

• *future medication doses *$\left\{{\left[PI\hat{D}OSE\;AI\hat{D}OSE\right]}_{k+j},j=\;0,\;.\;.\;.\;,\;c-1\right\}$ are doses that are obtained by the optimization algorithm assuming constant doses from *k *+ *c *to *k *+ *p − *1 set to the values of. ${\left[PI\hat{D}OSE\;AI\hat{D}OSE\right]}_{k+c-\text{1}}$

At the k-th time point, values of medication doses at the next *c *time points are calculated to minimize regularized predicted deviations from the reference trajectory over the prediction horizon while satisfying the constraints

(7) $$\underset{AIDOSE,PIDOSE}{\text{min}}\overset{p}{\underset{j=1}{\sum }}\left(w_{D}{\hat{D}}_{k+j}^{2}+w_{P}{\hat{P}}_{k+j}^{2}\right)+w_{c}\overset{p}{\underset{j=1}{\sum }}\left(PI\hat{D}OSE_{k+j-1}^{2}+AI\hat{D}OSE_{k+j-1}^{2}\right),$$

subject to

$$0\leq AI\hat{D}OSE_{k+j-\text{1}}\leq AIDOSE_{k+j-\text{1}}^{MAX},$$

$$0\leq PI\hat{D}OSE_{k+j-\text{1}}\leq PIDOSE_{k+j-\text{1}}^{MAX},$$

where *w_D_*, *w_P_*, and *w_c _*are weighting constants. Then, the first doses $AI\hat{D}OSE_{k}$ and $PI\hat{D}OSE_{k}$are applied to a virtual patient model to obtain new patient state at *k *+ 1. At the next time point *k *+ 1, the optimization procedure is repeated and a new sequence of doses is obtained.

Finding successful treatment by applying two medications is a challenging problem. Applying a large amount of *AIDOSE *would increase the level of *CA*, which would reduce the level of *D*. On the other hand, this might cause extreme growth of pathogen *P*. Also, applying a large amount of *PIDOSE *would increase the level of *N*, which would reduce the level of *P*. However, this can trigger pro-inflammatory response to focus on eliminating *P*, after which the level of tissue damage *D *could not be controlled anymore. Thus, both medication doses and their timing are critical for successful treatment.

### Predictive model

Accuracy of the predictive model over the prediction horizon directly affects the quality of model predictive control. Design of our data-driven predictive model involves two steps: 1) specification of model structure 2) learning model parameters from training data. These steps will be further explained in following paragraphs. For the sake of clarity we named our predictive model as *DD-MPC*.

#### Structure of DD-MPC predictive model

We propose a predictive model that is composed of four sub-models, each assigned to one of the outputs *P*, *N*, *D*, and *CA*. We use the same set of input variables in all sub-models. This set includes past patient states as well as past medication doses. Let us denote patient's state at time point *k *as **y***_k _*and medication doses as **u***_k_*

(8) $${\text{y}}_{k}=\left[P_{k}\;N_{k}\;D_{k}\;CA_{k}\right]T$$

(9) $${\text{u}}_{k}=\;\left[AIDOSE_{k}\;PIDOSE_{k}\right]T$$

Functional form of the sub-model responsible for predicting $\hat{P}$over prediction horizon given patient's states and medication doses up to *k *can be written as

(10) $${\hat{P}}_{k+j}=F_{P}\left({\hat{y}}_{k+j-\text{1}},...,{\hat{y}}_{k+\text{1}},y_{k},...,y_{k+j-n_{py}},{\hat{u}}_{k+j-\text{1}},...,{\hat{u}}_{k},u_{k-\text{1}},...,u_{k+j-n_{pu}},\beta_{P}\right),$$

where *j *= 1,*..*., *p*; *F_P _*is a function with unknown parameters ***β***_*P*_; *n_py _*and *n_pu _*are time lags. Other sub-models responsible for prediction of *N*, *D*, and *CA *have similar functional forms, but with different parameters ***β***_*N*_, ***β***_*D*_, and ***β***_*CA*_.

The next step is to define input-output functional relations *F_P_*, *F_N_*, *F_D_*, or *F_CA _*in terms of model parameters. Data driven non-linear predictive models such as Gaussian processes [12], neural networks [13], particle filters [14] have been successfully developed for industrial applications, although designing non-linear models in control theory is difficult task [15]. Also, non-linear models require much more data to be trained then linear models. Moreover, in the case of multivariate process, the difference in necessary training data is even larger. Due to high cost and limited availability of clinical data we applied linear functional forms in terms of ***β***_*P*_, ***β***_*N*_, ***β***_*D*_, ***β***_*CA*_.

#### Learning of DD-MPC predictive model

To follow a real life scenario regarding the availability of representative training data, we assume that the data come from a small population of diverse patients. To generate training data we select *N_tr _*virtual patients represented by ODE and consider that hourly measurements are available for a whole week. During the data generation process, initial conditions and parameters for each patient are randomly chosen from valid ranges while dosages are determined as follows.

In spite of control theory where control signals are randomly generated, random generation would not be clinically relevant for medication doses and thus we designed the following approach. We use MPC for each patient in the training set, deploying its own ODE as a predictive model. In this setup, the predictive model provides perfect predictions for patient's future states because predictions and future observations are the same at every time point. Therefore, by applying MPC we obtain ideal medication dosage. Ideal dosage is neither realistic in clinical practice nor suitable for learning data-driven models. Therefore, we added random Gaussian noise to the ideal sequence of doses, obtaining dosage that is close-to-ideal. On the other hand, close-to-ideal doses applied every hour over an entire week would lead many patients to a non-healthy state. Instead, close-to-ideal dosage strategy is applied in the first 10 hours of therapy (the most critical period) and then therapy is continued with the ideal doses. This strategy is called close-to-ideal dosage strategy.

## Evaluation

### Parameters of DD-MPC

In order to evaluate DD-MPC we need to decide on time lags, prediction horizon, and control horizon. The model with time lags equal to 2 had significantly larger predictive accuracy than the models with time lags different from 2. Therefore we use the model with time lags set to 2 in all of our experiments. The duration of the prediction horizon had to be set to achieve an equilibrium between following requirements: (1) the duration should be long enough in order to allow the therapy to fully manifest, (2) the duration should be short enough since predictive models are usually less reliable as the duration increases. The best performance was achieved with the length of prediction horizon window set to 5. The longer duration of control horizon makes response to be faster, but the system then becomes susceptible to model uncertainties [15]. Therefore we set the window length of control horizon to 2. Weights *w_P_*, *w_D_*, and *w_c _*in objective function were set to 3, 1, and 1 respectively in order to amortize the effect of unequal scalings.

### Number of patients to train DD-MPC

Obtaining the data is expensive in clinical applications. Hence, it is important that we investigate the least number of patients who are needed for the training data set so that the model learned on such data achieves acceptable accuracy. We generated a population of 65 virtual patients with the close-to-ideal dosage strategy for each of them. Each patient was observed for 168 hours and data were recorded on hourly bases. Finally, there were 43 healthy, 14 aseptic, and 8 septic patients' outcomes. It is worth noting that during training of the proposed data-driven model we did not take into account any prior knowledge about the data generation process.

We sampled *N_tr _*patients from population of 65 patients to train our predictive model with the assumption of equal number of healthy, aseptic, and septic patients in the sample. In order to determine minimal *N_tr _*for which predictive model gives acceptable accuracy, we evaluated percentage of healthy outcomes for MPCs with predictive models trained on varying number of *N_tr _*= 3, 6, 9, . . . , 24 patients. The validation of these models was performed on a population of 50 patients generated separately from the population used in training. The average number of healthy, aseptic, and septic outcomes for each *N_tr _*is presented in Table 1 together with standard deviation from 10 repeated samplings. From Table 1 we can see that models trained on a small number of patients are not successful in finding MPC based treatment. The balance between number of aseptic and septic outcomes and number of patients in training set was achieved for *N_tr _*= 18. Also, for *N_tr _*= 18 the result is stable over 10 repeated experiments with low standard deviation which suggest that for *N_tr _*= 18 the performance does not depend on the specific patients selected for training. Therefore, in next sections we use a *DD-MPC *model trained on 18 patients.

**Table 1:** Average number of healthy, aseptic and septic patients after *DD-MPC *therapy on a set of 50 patients.

*N_tr_*	Healthy	Aseptic	Septic
3	24.1 *± *9.7	12.7 *± *9.0	13.2 *± *7.4
6	26.6 *± *12.1	10.0 *± *5.8	13.4 *± *9.2
9	37.8 *± *11.1	7.4 *± *4.7	4.8 *± *8.1
12	41.7 *± *3.5	6.0 *± *2.0	2.3 *± *4.6
15	41.7 *± *3.2	5.8 *± *2.3	2.5 *± *5.0
18	44.1 *± *1.1	5.8 *± *0.7	0.3 *± *1.0
21	44.3 *± *1.2	5.3 *± *0.5	0.4 *± *1.2
24	43.6 *± *0.8	5.9 *± *0.9	0.5 *± *1.2

Predictive model was learned on *N_tr _*patients.

### Dataset

The proposed *DD-MPC *was evaluated on a population of 500 virtual patients recorded for 168 hours (1 week) by hourly measurements of *P*, *N*, *D*, and *CA*. In the case when treatment is not applied, the population has 85 septic, 117 aseptic, and 298 healthy virtual patients. In order to determine which patients require treatment, we follow the criteria from [6]. According to [6], if *N *exceeds 0.05 at any time point, then the corresponding patient needs to receive treatment. We found 321 out of 500 patients who were supposed to be treated. These patients represent a population on which we compare *DD-MPC *and baseline methods.

### Baseline methods

We compare therapy outcomes of our *DD-MPC *to models used in [6]:

• no therapy applied model (*Placebo*);

• the therapy currently used in the clinical practice: a constant anti-inflammatory therapy; we simulated this kind of therapy by applying *AIDOSE *= 0.005 hourly during the fist 72 hours from therapy onset (*N >*0.05), after 72 hours the therapy was terminated (*Static*);

• therapy based on MPC that used the mathematical model which parameters were fixed to parameters from a single patient [6] (*Mismatch*).

### Results

Therapy outcome was classified based on patient state at the end of 168 hours of simulation. If the patient did not reach stable state after 168 hours, then the simulation was extended for an additional 300 hours with no therapy applied. We report percentage and number of septic, aseptic and healthy outcomes at the end. In addition, we distinguish different groups of patients based on the effect of therapy:

• harmed - designates cases when the outcomes of therapy without using any medications (*Placebo*) were healthy, while outcomes after applying therapy were aseptic or septic (the lower percentage of harmed the better);

• rescued - designates cases when outcomes after therapy were healthy, while outcomes without therapy were aseptic or septic (the higher percentage of rescued the better).

#### Fully observed data

Results of treatments using *DD-MPC *and baselines on fully observed patients who were assigned to receive therapy are reported in Table 2. *DD-MPC *achieved around 8.5% higher percentage of healthy outcomes than *Mismatch *and around 50% higher than *Placebo *and *Static*. At the same time *DD-MPC *managed to keep 0 harmed patients out of 119 for whom the outcome of *Placebo *therapy was healthy. In addition, application of *DD-MPC *rescued 87% of 202 patients that would otherwise be aseptic or septic within a 168 hours period. In contrast, *Mismatch *rescued only 73% of patients.

**Table 2:** Number and fraction of patients on fully observed data for: model with no therapy applied (*Placebo*), model with constant anti-inflammatory dose (*Static*), MPC with mathematical predictive model with set of parameters equal to parameters of a single patient (*Mismatch*) and MPC with data-driven predictive model learned on small data sample (*DD-MPC*) and MPC with data-driven predictive model learned on small data sample with 5% additive Gaussian noise in observations (*DD-MPC+noise*).

	Healthy (total 321)	Aseptic (total 321)	Septic (total 321)	Harmed (total 119)	Rescued (total 202)
*Placebo*	119 (37.07%)	117 (36.45%)	85 (26.48%)	N/A	N/A
*Static*	140 (43.61%)	96 (29.91%)	85 (26.48%)	3 (2.52%)	24 (11.88%)
*Mismatch*	267 (83.18%)	50 (15.58%)	4 (1.25%)	0 (0%)	148 (73.27%)
*DD-MPC*	294 (91.59%)	24 (8.41%)	0 (0%)	0 (0%)	175 (86.63%)
*DD-MPC*+*noise*	284 (88.47%)	33 (10.28%)	4 (1.25%)	0 (0%)	165 (81.68%)

**Figure 3:**
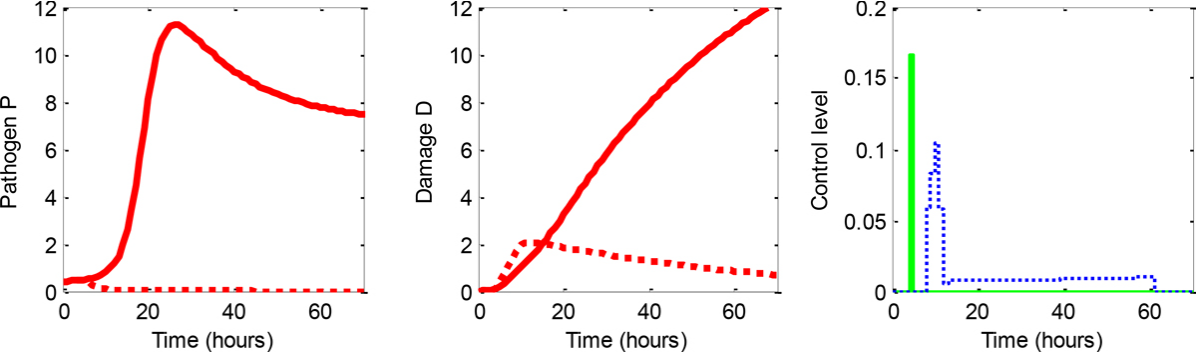
**An example of successful optimal therapy found by *DD − MPC*: *Placebo *(solid red) and *DD −MPC *(dashed red)**. Dosage found by *DD − MPC*: *AIDOSE *(solid green) and *PIDOSE *(dashed blue).

In Figure 3 we illustrated an example of a therapy common for the rescued virtual patients. As we can see, at the early stadium of acute inflammation a high pro-inflammatory medication was applied in order to fight against invasive pathogen. Once the pathogen level was reduced, the therapy continued by applying anti-inflammation medication to reduce inflammation and recover the patient to healthy state.

To identify patients (represented by initial conditions and model parameters) who benefit the most from using *DD-MPC*, we compare the outcomes of the *DD-MPC *model to the outcomes of *Mismatch*. According to possible outcomes, we split patients into two groups: (1) 266 patients who were successfully treated by both models, and (2) 33 patients who were successfully treated by *DD-MPC *but not *Mismatch*. The group of 33 patients is characterized by high initial level of pathogen *P*_0 _(*P*_0 _= 0.78 *± *0.13). In order to detect the conditions for which *DD-MPC *outperforms *Mismatch*, we compare 33 patients to a subset of 165 patients from another group who had similar *P*_0 _(*P*_0 _= 0.79 *± *0.12). Average values of parameters *k_cn _*and *k_nd _*were significantly different between the group of 33 patients and the subset of 165 patients according to two tailed t-test with 95% confidence. We discovered that the *Mismatch *mathematical model had similar values of *k_cn _*and *k_nd _*to the ones found in the subset of 165 patients. On the other hand, the difference in the parameter values of *Mismatch *and the group of 33 patients affected predictive power of *Mismatch *on these patients and thus it was not able to provide adequate therapy. Oppositely, data driven *DD-MPC *generalized well what it learned on training data which resulted in succesful treatment outcomes on both groups of patients.

#### Therapy efficiency

Therapy efficiency was evaluated by the average area-under-the-curve (AUC) of pathogen level *P *and tissue damage *D *over the patients with healthy outcome that were assigned to the treatment. The lower the AUC is, the more effective therapy is. *DD-MPC *outperformed *Mismatch *by achieving lower average AUC(*P*) and lower average AUC(*D*) (Table 3). We also compared the average AUC of *PIDOSE *and *AIDOSE *used per healthy outcome. Drugs have potential harmful side effects as well as financial costs, so the lower usage the better, but only if it does not affect the quality of therapy. From Table 3 we see that *DD-MPC *by using lower dosage, achieved a higher percentage of healthy outcomes than *Mismatch*.

**Table 3:** Comparison of therapy strategies with respect to average per healthy patient of: area under curve (AUC) of pathogen level *P*, AUC of tissue damage *D*, anti-inflammatory therapy *AIDOSE*, and pro-inflammatory therapy *PIDOSE *(lower score is better).

	AUC(*P*)	AUC(*D*)	*PIDOSE*	*AIDOSE*
*Mismatch*	4.56	182.23	0.3053	0.8301
*DD-MPC*	4.39	147.46	0.2261	0.7814

#### Delay in diagnosis

**Figure 4:**
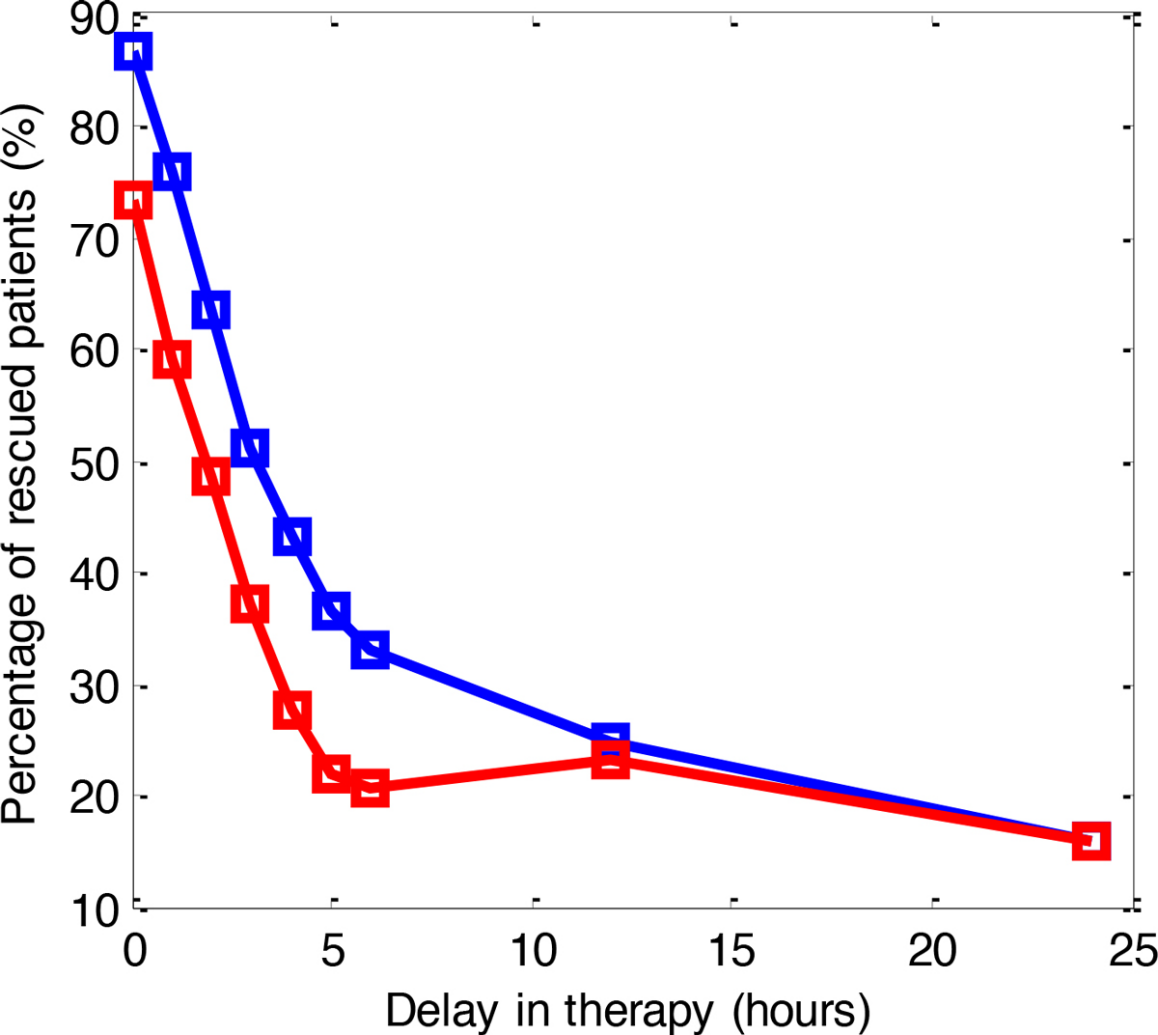
**The effect of therapy delay of up to 24 hours from a threshold-based decision (*N >*0.05)**. Percentage of rescued patients by DD-MPC (blue) vs. Mismatch (red) therapies.

To show the importance of timely diagnosis of acute inflammation, we performed an experiment where we delayed the onset of a therapy and measured the effect of the delay on final outcome. In particular, instead of initiating therapy when *N *reached 0.05 we initiated it with 1, 2, . . . 24 hours delay. Figure 4 shows the effect of the delays on final outcomes when *DD-MPC *and *Mismatch *were applied. The *DD-MPC *outperforms *Mismatch *for shorter delays while for longer delays both methods are equally good. The percentage of rescued patients of *DD-MPC *decreases at a rate of about 12% per hour in first few hours after acute inflammation was identified. This simulation result suggests that acute inflammation therapy requires timely application in order to be effective for treatment.

#### Noisy data

Since measurement noise is inevitable, we tested the robustness of *DD-MPC *to additive 5% Gaussian noise (standard deviation is 5% of the measurement) that influenced each hourly measurement of all four outputs. From Table 2 we can notice that there was no significant decrease in results compared to the results obtained with an ideal noise free system.

#### Partially observed data

We considered the clinically relevant scenario that only incomplete measurements are available over time (we assumed that complete data was only available for training). We followed the approach from [6] by assuming that hourly measurements for *N *and *CA *were available at any time. Level of tissue damage *D *was impossible to quantify at all, while an indirect measurement of the pathogen level *P *was available every four hours. Every four hours, pathogen levels predicted by the predictive model were compared to pathogen levels obtained from the patient model. When pathogen level in the predictive model was significantly different than pathogen level in the patient model then: (1) if pathogen level in the predictive model was lower than pathogen level in the patient model, then the predictive model's pathogen level for the next step was reset to *P *= 0.5, or (2) if pathogen level in the predictive model was higher than pathogen level in the patient model then the predictive model's pathogen level was reset to zero. As suggested in [6], the use of the pathogen levels in this way was clinically relevant, reflecting the fact that in a clinical setting an infection can be identified based on other parameters, such as high fever. On the evaluation data, *DD-MPC *used predictions of *P *and *D *from a previous time step as inputs in the next time step, confirming the assumption that these values are not observable. Results are presented in Table 4. Although accuracy decreases compared to the case when all measurements were available, *DD-MPC *with partially observed measurements achieved better results than *Placebo *and *Static *from Table 2 and was comparable to *Mismatch *that used all variables. *Mismatch *accuracy also decreased when compared to accuracy on fully observed data.

**Table 4:** Number and fraction of healthy, aseptic and septic patients on partially observed data for: MPC with mathematical predictive model with set of parameters equal to parameters of a single patient (*Mismatch*) and MPC with data-driven predictive model learned on small data sample (*DD-MPC*).

	Healthy (total 321)	Aseptic (total 321)	Septic (total 321)	Harmed (total 119)	Rescued (total 202)
*Mismatch*	250 (77.88%)	59 (18.38%)	12 (3.74%)	2 (1.68%)	133 (65.84%)
*DD-MPC*	267 (83.18%)	12 (3.74%)	42 (13.08%)	0 (0%)	148 (73.27%)

## Conclusion

We presented a data-driven method for acute inflammation therapy (*DD-MPC*), which was evaluated through a number of experiments performed on virtual patients. We demonstrated that a population of 18 healthy, aseptic and septic patients was enough to learn an efficient data-driven predictive model. Obtained results showed that *DD-MPC *outperformed clinically relevant alternatives, providing good results even in the presence of incomplete measurements as well as additive Gaussian noise. Furthermore, the therapy based on *DD-MPC *did not harm any of the healthy patients, which is a property of high importance when the method is applied in practice. Finally, promising results presented in this paper provide evidence that research on acute inflammation treatment can benefit from methods from the machine learning community.

## Competing interests

The authors declare that they have no competing interests.

## Authors' contributions

VR and KR contributed equally to this work. VR and KR conceived of the study, participated in its design, performed the statistical analysis, and wrote the manuscript. ZO conceived of the study, oversaw its design and coordination, and participated in interpretation of the analyses and writing of the manuscript. All authors read and approved the final manuscript.

## Declarations

The publication costs for this article were funded by the corresponding author.

This article has been published as part of *BMC Medical Genomics *Volume 6 Supplement 3, 2013: Selected articles from the IEEE International Conference on Bioinformatics and Biomedicine 2012: Medical Genomics. The full contents of the supplement are available online at http://www.biomedcentral.com/bmcmedgenomics/supplements/6/S3.
